# Functional CCR7A-mediated cellular responses are negatively modulated by the splice variant CCR7B

**DOI:** 10.1371/journal.pone.0354819

**Published:** 2026-08-04

**Authors:** Minyeong Cho, Hee-Kyung Park, Lan Phuong Nguyen, Thai Uy Nguyen, Duc Trung Nguyen, Soyeon In, Sunghoon Hurh, Beom Jin Park, Jae Young Seong, Jong-Ik Hwang

**Affiliations:** 1 Department of Biomedical Sciences, College of Medicine, Korea University, Seoul, Republic of Korea; 2 Department of Radiology and Advanced Medical Imaging Institute, College of Medicine Anam Hospital, Korea University, Seoul, Republic of Korea; Uniformed Services University, UNITED STATES OF AMERICA

## Abstract

C-C chemokine receptor 7 (CCR7) directs immune cell homing to secondary lymphoid organs and has been implicated in cancer metastasis through its ligands CCL19 and CCL21. Human CCR7 pre-mRNA undergoes alternative splicing to generate five transcripts that encode three protein isoforms with distinct N-termini, termed CCR7A, CCR7B, and CCR7C, but their comparative properties and cross-regulation are not well defined. Here, we cloned these three isoforms and systematically characterized their expression, localization, signaling, and mutual interactions in mammalian cells under both strong (CMV) and weaker (ubiquitin C, UbiC) promoter control to reduce overexpression-related artifacts. Variant-specific RT-PCR revealed that transcripts encoding CCR7A (V1) and CCR7B (V2) predominate in diverse human cell lines, whereas CCR7C-encoding variants (V3–V5) are weakly expressed. EGFP imaging and HiBiT-based assays showed efficient plasma-membrane targeting of CCR7A, partial membrane localization and prominent perinuclear accumulation of CCR7C, and largely cytosolic retention of CCR7B. Under UbiC-driven expression, CCR7A mediated robust CCL19- and CCL21-induced Gi/o and Gq-like activation, intracellular Ca² ⁺ mobilization, ERK phosphorylation, GRK3-dependent Gβ1 recruitment, and β-arrestin1 binding, whereas CCR7C displayed weaker and mainly CCL19-biased signaling. CCR7B did not respond to either chemokine in any signaling readout and thus behaved as a non-signaling isoform. NanoBiT-based assays and co-immunoprecipitation demonstrated that all three isoforms form homo- and heterodimers, with particularly strong association between CCR7A and CCR7B. Co-expression of CCR7B reduced CCR7A surface expression and markedly attenuated chemokine-induced Ca² ⁺ responses, mini-Gi interaction, and β-arrestin1 recruitment, while confocal microscopy revealed redistribution of CCR7A-EGFP from the plasma membrane to intracellular compartments. Moreover, MDA-MB-231 breast cancer cells, which express CCR7A and CCR7B transcripts, did not migrate toward CCL19 or CCL21 despite preserved motility toward low-serum medium. These findings identify CCR7A as the dominant functional isoform, CCR7C as a weak CCL19-biased receptor with inefficient membrane targeting, and CCR7B as a non-signaling dominant-negative isoform that dampens CCR7A-mediated responses, suggesting that CCR7 splicing fine-tunes chemokine responsiveness in immune and cancer cells.

## Introduction

Chemokines are small secreted cytokines that control the migration and positioning of immune cells under homeostatic and inflammatory conditions by signaling through G protein-coupled receptors (GPCRs) [1–3]. CCR7 is a prototypical homeostatic chemokine receptor that recognizes CCL19 and CCL21 and is primarily expressed in naive T cells, some B cells, and mature dendritic cells [4,5]. By guiding these cells to secondary lymphoid organs via constitutive chemokine gradients, CCR7 plays a critical role in immune surveillance and tolerance [6,7].

Beyond physiological immune cell trafficking, CCR7 has been implicated in various pathologies, including autoimmune diseases and cancer [8–10]. In several malignancies, such as breast and colorectal cancer, elevated CCR7 expression has been associated with enhanced lymph node metastasis and poor prognosis, presumably by enabling tumor cells to migrate along CCL19/CCL21 gradients toward draining lymph nodes [11,12]. Regarding to immune cell mobilization, CCR7 inhibition may alleviate inflammation in autoimmune diseases such as rheumatoid arthritis by reducing cell number in damaged tissues [13,14]. However, global inhibition of CCR7 carries a risk of severe immunological side effects because of its central role in lymphocyte homing, underscoring the need to better understand how CCR7 signaling is regulated [15,16].

Alternative splicing is an important mechanism that diversifies the GPCR repertoire and can generate receptor isoforms with distinct trafficking and signaling properties [17,18]. Many chemokine receptors have relatively simple single-exon coding regions in rodents, but more complex exon–intron structures and alternative splicing patterns in humans. In several cases, splicing affects the N-terminal extracellular region, which is critical for chemokine binding, signal peptide function, and cell surface targeting. Splice variants of receptors such as CCR2 and CXCR3 differ in membrane expression, ligand specificity, and β-arrestin-dependent signaling, highlighting the regulatory potential of this mechanism [19,20].

The human CCR7 gene yields at least five mRNA splice variants (V1–V5) that encode three protein isoforms with distinct N-terminal regions, designated CCR7A, CCR7B, and CCR7C. CCR7A corresponds to the longest isoform and has generally been considered the canonical receptor. CCR7C is produced from transcripts using an alternative first exon and a downstream start codon, leading to a shorter signal peptide and N-terminus. CCR7B arises from transcripts that skip an upstream exon and initiate translation from a more downstream AUG within a large common exon, resulting in a receptor essentially lacking the conventional N-terminal extracellular domain. Although these isoforms are annotated in genomic databases, their comparative expression, signaling capacity, and functional interactions have not been comprehensively investigated.

Most prior functional studies of chemokine receptors have relied on strong viral or CMV promoters, which induce supra-physiological receptor expression and can exaggerate signaling and dimerization, masking more subtle differences between splice variants [21]. To more closely approximate physiological conditions and reduce overexpression-related artifacts, we combined such systems with a weaker ubiquitin C (UbiC) promoter that supports lower, more controlled expression.

In this study, we cloned the three human CCR7 protein isoforms and evaluated (i) their gene structure and N-terminal sequence features; (ii) isoform-specific transcript expression patterns in multiple human cell lines; (iii) subcellular localization and surface expression; (iv) ligand-induced coupling to mini-G proteins and intracellular Ca² ⁺ mobilization; (v) ERK phosphorylation, GRK recruitment, and β-arrestin1 binding; (vi) homo- and heterodimer formation; and (vii) the impact of CCR7B co-expression on CCR7A function, including in a metastatic breast cancer cell line. Our data identify CCR7A as the main functional receptor, CCR7C as a weak CCL19-biased isoform, and CCR7B as a non-signaling dominant-negative modulator of CCR7A-mediated responses.

## Materials and methods

### Materials

Chemokines were purchased from Peprotech (Rocky Hill, NJ, USA). NanoBiT® PPI starter system and Nano-Glo® live cell assay system; pBiT3.1 plasmid and Nano-Glo® HiBiT extracellular detection system were purchased from Promega (Madison, WI, USA). Anti-ERK antibody (Cat. No. 4695) and anti-pERK antibody (Thr202/Tyr204) (Cat. No. 4370) were purchased from Cell Signaling Technology (Beverly, MA, USA). Anti-myc- tag antibody (Cat. No. 2278) and anti-HA agarose (Cat. No.26181) for co-IP were purchased from cell signaling Technology (Beverly, MA, USA) and Thermo Scientific (Waltham, MA, USA) respectively. Anti-Green fluorescence protein (GFP) antibody (Cat. No. sc-8334), anti-β-actin antibody (Cat. No. sc-9996), and all secondary antibodies were purchased from Santa Cruz Biotechnology (Santa Cruz, CA, USA). All primers for gene cloning and PCR and related materials were obtained from Cosmo Genetech Co., Ltd. (Seoul, Korea) and the DNA sequencing was conducted by Cosmo Genetech Co., Ltd. (Seoul, Korea). Restriction enzymes were purchased from New England Bio Labs (Ipswich, MA, USA).

### Plasmid construction

Human CCR7 splice variants encoding CCR7A (V1), CCR7B (V2), and CCR7C (V3–V5) were amplified by PCR from human cDNA using variant-specific primers and cloned into pcDNA3.1-based vectors. To enable controlled expression, the CMV promoter in pcDNA3.1 was replaced by the human ubiquitin C (UbiC) promoter, and each CCR7 coding sequence was inserted into these modified vectors. For comparison, CMV-driven constructs were also generated. C-terminal HA, EGFP, SmBiT, or LgBiT tags were introduced by standard subcloning. For HiBiT assays, HiBiT was placed either at the extreme N-terminus or immediately downstream of the predicted signal peptide of CCR7A and CCR7C. All constructs were verified by Sanger sequencing.

### Cell culture

HEK293 and MDA-MB-231 cells were obtained from the American Type Culture Collection and cultured in Dulbecco’s modified Eagle’s medium supplemented with 10% fetal bovine serum, 100 U/ml penicillin, and 100 μg/ml streptomycin at 37°C in a humidified 5% CO_2_ incubator. Additional human cell lines used for RT-PCR analysis (HUVEC, A549, HepG2, KG-1, U937, Jurkat, Raji, PC-3) were maintained according to the supplier’s recommendations. A HEK293-derived cell line stably expressing a chimeric Gqi protein was used for Ca² ⁺ mobilization assays.

### RT-PCR for variant-specific expression

Total RNA was isolated from cell lines using TRIzol Reagent (Invitrogen, Carlsbad, CA, USA) following the manufacturer’s protocol. The extracted RNA was then reverse transcribed into cDNA using M-MLV reverse transcriptase and random hexamers. RT-generated cDNA encoding CCR7 splice variants was amplified through 35 cycles of PCR under the following conditions: 95°C for 30 seconds, 58°C for 30 seconds, and 72°C for 30 seconds, with a final extension at 72°C for 10 minutes. PCR was carried out using a SimpliAmp Thermal Cycler (Thermo Fisher Scientific). The primers used for amplification were as follows: CCR7 V1 (CCR7A): Forward 5’- GTCATGGACCTGGGGAAACCAATG −3’, Reverse 5’-GAAGTAGGAGCATGCCACTGAAG-3’; V2 (CCR7B): Forward 5’- GAGCGTC ATGGACCTGGGTATGC −3’, Reverse 5’-GAAGTAGGAGCATGCCACTGAAG-3’; V3-5 (CCR7C): Forward 5’-GAGCCCCTGAGGATTTAGGAGG-3’, Reverse 5’-GAAGTAGGAGCATGCCACTGAAG-3’. PCR products were separated on 1.5% agarose gels and visualized with ethidium bromide. Band sizes were compared with predicted fragment lengths for each variant.

### Western blotting and co-immunoprecipitation

HEK293 cells transiently expressing CCR7 variants were serum-starved overnight and stimulated with 100 ng/ml CCL19 or CCL21 for 5 min for ERK phosphorylation. Cells were lysed with lysis buffer (150 mM NaCl, 50 mM Tris-HCl pH7.5, 10 mM KCl, 1% Triton X-100, 10 mM NaF, 5 mM Na_3_VO_4_) and protease inhibitor cocktail (Roche, Indianapolis, IN, USA). Proteins were separated by SDS-PAGE and transferred to nitrocellulose membranes. Membranes were probed with antibodies against ERK1/2 and phospho-ERK1/2 followed by HRP-conjugated secondary antibodies and chemiluminescent detection with an enhanced chemiluminescence (ECL) reagent (Thermo Scientific, Rockford, IL, USA).

For co-immunoprecipitation, HEK293 co-expressing myc-his-tagged CCR7A/B/C and HA-tagged CCR7A were lysed and incubated with anti-HA agarose. After washing, bound proteins were eluted and analyzed by Western blotting with anti-myc antibody (Cell Signaling Cat. No. 2278). Samples used for CCR7 detection were not boiled to preserve receptor integrity.

### Confocal imaging

HEK293 cells grown on poly-L-lysine-coated coverslips were transfected with C-terminal EGFP-tagged CCR7A, CCR7B, or CCR7C. Twenty-four hours after transfection, cells were fixed with 4% paraformaldehyde and counterstained with DAPI. EGFP fluorescence was observed by fluorescence or confocal microscopy. For co-localization, cells were co-transfected with CCR7A-EGFP and CCR7B-tRFP and imaged under the same conditions.

### HiBiT-based surface expression assay

To quantify cell surface expression, HEK293 cells in 96-well plates were transfected with increasing amounts of HiBiT-tagged CCR7A/B/C plasmids. After 24 h, cells were incubated with Nano-Glo HiBiT extracellular detection reagent according to the manufacturer’s protocol, and luminescence was measured with a plate reader. For constructs in which HiBiT was placed downstream of the signal peptide of CCR7A or CCR7C, the same procedure was followed. For co-expression experiments, HiBiT-CCR7A was transfected with increasing amounts of CCR7B plasmid.

### NanoBiT-based protein-protein interaction assays

NanoBiT assays were used to monitor (i) interactions between CCR7 isoforms and mini-G proteins (Gs, Gsi, Gsq, Gs12), (ii) interactions between Gβ1 and GRK3 or GRK6, (iii) recruitment of β-arrestin1 to CCR7 isoforms, and (iv) homo- and heterodimerization among CCR7 isoforms. HEK293 cells were co-transfected with SmBiT- and LgBiT-tagged constructs under UbiC promoter control. After 24 h, cells were equilibrated in Opti-MEM, incubated with Nano-Glo Live Cell substrate, and basal luminescence was recorded. Cells were then stimulated with 100 ng/ml CCL19 or CCL21, and luminescence was monitored for up to 60 min. For dose–response curves, increasing ligand concentrations were applied, and peak responses were used to estimate EC50 values.

### Ca² ⁺ mobilization assay

Gqi-expressing HEK293 cells were co-transfected with CCR7 variants and NanoBiT-based Ca² ⁺ probes consisting of SmBiT-tagged calmodulin and LgBiT-MYLK2S carrying a calmodulin-binding sequence, which were previously constructed [22]. After 24 h, cells were incubated in Opti-MEM, treated with Nano-Glo Live Cell substrate, and baseline luminescence was recorded. Cells were then stimulated with CCL19 or CCL21, and time-dependent luminescence changes were measured. Responses were normalized to basal levels to calculate fold increases. CMV- and UbiC-driven constructs of the receptors were examined to compare expression efficiency-dependent responses to the chemokines.

### Chemotaxis assay

Chemotaxis was assessed using 24-well Transwell plates with 8-μm pore size inserts. MDA-MB-231 cells were seeded in the upper chamber in serum-free medium containing 0.1% BSA. The lower chambers contained serum-free medium with 0.1% BSA supplemented with 100 ng/ml CCL19, 100 ng/ml CCL21, or 0.2% FBS as a positive control. After 24 h, non-migrated cells were removed from the upper side of the membrane, and migrated cells on the lower side were fixed, stained with hematoxylin and eosin, and counted in multiple high-power fields. CCR7A and CCR7B genes were inserted into FG12 lentiviral vector which contains UbiC promoter for gene expression. The virus was produced by transfecting the viral vector and the accessory vectors into HEK293T cells using calcium phosphate. Jurkat cells expressing exogenous CCR7A or CCR7A/CCR7B were subjected to the Transwell migration assay. After 6 h, the media in the lower chamber were collected and the migrated cells toward CCL19 were counted.

### Flow cytometry

Jurkat cells and trypsinized MDA-MB231 cells were resuspended in phosphate buffered saline (PBS) containing 2% FBS at 5 × 10^5^ cells per experimental sample. Cells were stained with either PE-conjugated mouse IgG2a, κ Isotype Control antibody (BioLegend, Cat. No. 400213) or PE-conjugated anti-human CCR7 antibody (BioLegend, Cat. No. 353203) by incubating for 20 min at room temperature in the dark, using the manufacturer-recommended antibody dilution. Cells were then washed three times with PBS containing 2% FBS to remove unbound antibodies. Flow cytometric analysis was performed using a BD FACSLyric flow cytometer (BD Biosciences, USA), and acquired data were analyzed using FlowJo^TM^ software (Tree Star Inc., Ashland, OR, USA) excluding debris and doublets.

### Statistical analysis

Data are presented as mean ± SEM from at least three independent experiments unless otherwise indicated. Statistical significance was evaluated using unpaired Student’s t-tests or one-way ANOVA with Bonferroni’s multiple comparison test. P values < 0.05 were considered significant.

## Results

### N-terminal features and expression properties of CCR7 splice variants

**Fig 1:**
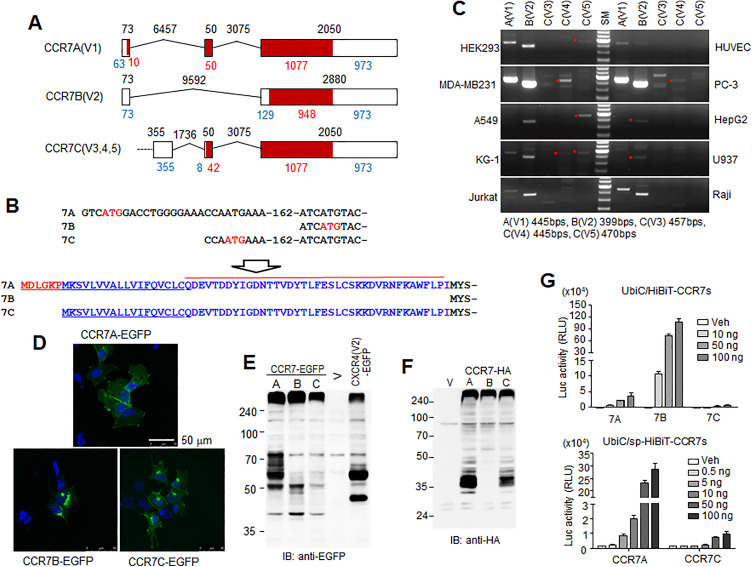
Expression patterns of CCR7 splice variants in human cells. (A) Schematic representation of the five human CCR7 mRNA splice variants (V1–V5). Red boxes indicate protein-coding regions within exons; white boxes, untranslated regions; black lines, introns. Black numbers denote nucleotide positions within exons or introns; red numbers, nucleotides belonging to the open reading frame (ORF); blue numbers, untranslated nucleotides within exons. (B) Alignment of the translation start sites and N-terminal extracellular regions of CCR7A, CCR7B, and CCR7C. Underlines indicate predicted signal peptides. Red letters in CCR7A mark amino acids that are absent from CCR7C. (C) Variant-specific RT-PCR analysis of CCR7 transcripts in various human cell lines. Red asterisks indicate specific but weak bands corresponding to CCR7C-encoding variants (V3–V5). (D) Subcellular localization of C-terminal EGFP-tagged CCR7A, CCR7B, and CCR7C in HEK293 cells. Fluorescence images were acquired by confocal microscopy. Scale bar, 50 μm. (E) Western blot analysis of HEK293 cells transiently expressing C-terminal HA-tagged CCR7A, CCR7B, or CCR7C. Blots were probed with an anti-HA antibody. (F) Western blot analysis of C-terminal EGFP-tagged CCR7 variants in HEK293 cells. CXCR4 variant 2 (CXCR4(V2)-EGFP) was used as a reference GPCR. Blots were probed with an anti-EGFP antibody. (G) Cell surface expression of CCR7 variants measured by HiBiT assay. HEK293 cells were transfected with increasing amounts of HiBiT-tagged CCR7 plasmids, and extracellular HiBiT activity was quantified. Upper graph, HiBiT fused to the extreme N-terminus of CCR7A, CCR7B, or CCR7C. Lower graph, HiBiT inserted immediately downstream of the signal peptide of CCR7A or CCR7C (sp-HiBiT-CCR7A/CCR7C). Data represent the mean ± SEM of three independent experiments.

Database analysis from Ensembl (https://www.ensembl.org/) and National Center for Biotechnology Information (https://ncbi.nlm.nih.gov) and cDNA sequencing confirmed that the human CCR7 gene produces five mRNA splice variants (V1–V5) that encode three protein isoforms, CCR7A, CCR7B, and CCR7C, differing in their N-terminal regions. In CCR7A (V1), the open reading frame spans three exons, and translation initiates from an AUG codon in the first exon, generating a receptor with a 24-amino-acid signal peptide. CCR7C is produced from V3–V5 transcripts, which share an alternative first exon and use a downstream AUG in the second exon, resulting in an 18-amino-acid signal peptide and a six-residue shorter N-terminus than CCR7A. CCR7B arises from V2 transcripts lacking the second exon present in V3–V5; translation initiates at a further downstream AUG within a large common exon, producing a receptor that essentially lacks a conventional N-terminal extracellular domain and signal peptide (Fig 1A, B). The predicted signal peptides of CCR7A and CCR7C match the unique CCR7 signal sequence described previously [23], whereas CCR7B begins with a short “MYS” motif that is unlikely to function as a signal peptide. The Kozak sequence surrounding the CCR7C start codon is suboptimal compared with CCR7A, suggesting that CCR7C translation may be less efficient under endogenous promoter conditions (Fig 1B).

Variant-specific RT-PCR across multiple human cell lines showed that V1 (CCR7A) and V2 (CCR7B) transcripts were detectable in all examined cell lines, including HEK293, HUVEC, A549, HepG2, KG-1, U937, Jurkat, Raji, MDA-MB-231, and PC-3, although their expression levels varied considerably among cell types. In contrast, V3-V5 (CCR7C-encoding) transcripts produced only weak or undetectable bands across all cell lines examined. These data indicate that CCR7A and CCR7B are likely to be the predominant isoforms in many human cells, while CCR7C is expressed at low levels (Fig 1C).

### Subcellular localization and surface expression of CCR7 isoforms

To compare subcellular localization, C-terminal EGFP-tagged CCR7 isoforms were expressed in HEK293 cells and imaged by fluorescence microscopy. CCR7A-EGFP localized predominantly to the plasma membrane, with only faint intracellular staining. CCR7C-EGFP was present at the cell surface but also showed strong perinuclear accumulation consistent with endoplasmic reticulum and early secretory compartments, indicating inefficient trafficking to the plasma membrane. In contrast, CCR7B-EGFP was largely confined to the cytoplasm, with diffuse intracellular fluorescence and minimal membrane localization (Fig 1D).

Western blotting of EGFP- or HA-tagged receptors showed that all three isoforms migrated primarily as high-molecular-weight species near the stacking gel, consistent with SDS-resistant aggregates common among chemokine receptors as shown in CXCR4(V2)-EGFP [24]. CCR7A and CCR7C also displayed bands at the expected monomeric size (~37–55 kDa, depending on the tag), whereas CCR7B mainly appeared as aggregated and fragmented forms (Fig 1E, F).

Cell surface expression was quantified using N-terminal HiBiT-tagged constructs driven by Ubiquitin C (UbiC) promoter. HiBiT-CCR7A produced luminescence signals that increased with plasmid dose, although the maximum signal was lower than those of CXCR4 and CXCR3 as described in our previous reports [24,25]. HiBiT-CCR7C generated significantly weaker signals than CCR7A, consistent with its partial intracellular retention. Interestingly, HiBiT-CCR7B yielded relatively high luminescence despite lacking a native signal peptide, suggesting that the HiBiT-tag may act as a surrogate signal for membrane targeting (Fig 1G, upper graph). The relatively high HiBiT surface signal observed for HiBiT-CCR7B likely reflects an artifact in which the appended N-terminal HiBiT sequence partially substitutes for the missing signal peptide, facilitating ER insertion and plasma membrane delivery that does not occur with native CCR7B; this interpretation is supported by the predominantly cytosolic localization of CCR7B-EGFP, in which no exogenous N-terminal sequence is present. To avoid this artifact, we created constructs in which HiBiT was inserted immediately downstream of the signal peptide of CCR7A or CCR7C. Under these conditions, surface expression of sp-HiBiT-CCR7A and sp-HiBiT-CCR7C increased with plasmid dose, but signals from CCR7C remained substantially lower than those from CCR7A, confirming that CCR7C’s shorter N-terminus and suboptimal start site compromise efficient membrane localization (Fig 1G, lower graph). Since the signal peptide is cleaved upon ER translocation, these differences in surface expression reflect the distinct biosynthetic efficiencies conferred by the CCR7A and CCR7C signal peptides, rather than differences in the mature receptor sequence, which is identical between the two isoforms.

### Ligand-induced signaling by CCR7 isoforms under controlled expression

**Fig 2:**
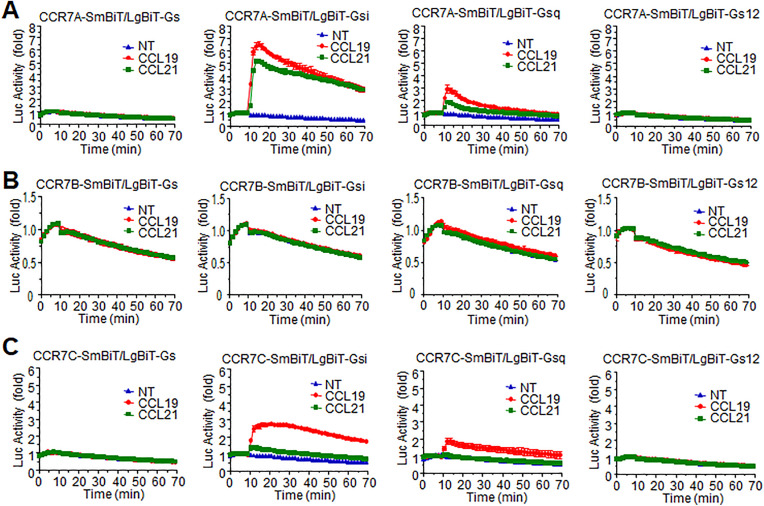
Chemokines-stimulated interaction between CCR7 variants and min-G protein constructs. C-terminal SmBiT-tagged CCR7 isoforms and N-terminal LgBiT-tagged mini-G constructs were co-expressed in HEK293 cells. Cells were stimulated with 100 ng/ml CCL19 or CCL21, and luminescence was recorded in real time as a measure of receptor–mini-G interaction. (A) CCR7A-SmBiT, (B) CCR7B-SmBiT, (C) CCR7C-SmBiT.

We next compared ligand-induced G protein coupling and Ca² ⁺ mobilization using NanoBiT-based mini-G assays and NanoBiT Ca² ⁺ sensors under UbiC- and CMV-driven expression. In cells expressing C-terminal SmBiT-tagged CCR7A and N-terminal LgBiT-mini-Gsi, both CCL19 and CCL21 rapidly increased luminescence to 6–8-fold above basal levels, indicating robust Gi/o activation. CCR7A also interacted with mini-Gsq, with 3–4-fold signal increases, whereas no significant interaction was observed with mini-Gs or G12. Thus, CCR7A primarily couples to Gi/o, with secondary engagement of Gq-like pathways (Fig 2A). As described in previous reports, mini-Gs12 construct may not be applicable to examine G12/13 interaction with the receptor, although many chemokine receptors mediate G12/13 pathways for cell migration toward their ligands [26,27].

CCR7C-SmBiT similarly exhibited ligand-induced interaction with mini-Gsi and mini-Gsq, but maximal responses were consistently smaller than those of CCR7A. Moreover, CCL19 elicited much stronger signals than CCL21, particularly for CCR7C, where CCL21 responses were minimal (Fig 2C). In contrast, CCR7B-SmBiT did not show any detectable ligand-induced interaction with mini-G proteins, indicating that it does not efficiently couple to G proteins under our conditions (Fig 2B).

**Fig 3:**
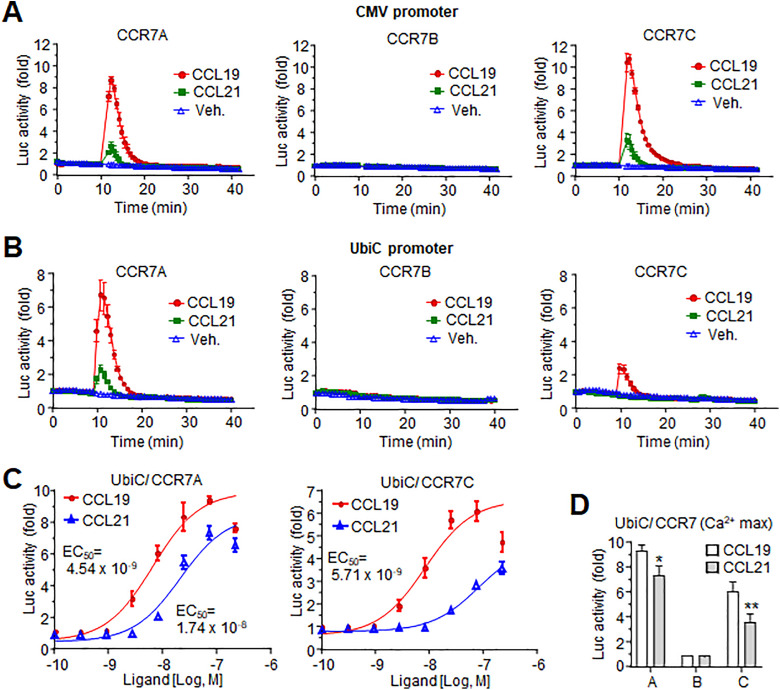
Chemokine-induced Ca² ⁺ signaling mediated by CCR7 variants. (A) HEK293 cells stably expressing Gqi were transiently transfected with CMV-driven CCR7 constructs together with NanoBiT Ca² ⁺ probes (SmBiT-fused calmodulin and LgBiT-MYLK2S). Cells were stimulated with 100 ng/ml CCL19 or CCL21, and luminescence was measured over time. (B) To evaluate receptor function under controlled expression, UbiC promoter-driven CCR7 constructs were co-transfected with the same Ca² ⁺ probes, and chemokine-induced luminescence changes were monitored. (C) Concentration-dependent Ca² ⁺ responses to CCL19 and CCL21 in cells expressing UbiC-driven CCR7A or CCR7C. Peak luminescence values were used to generate dose–response curves and estimate EC₅₀ values. (D) Maximal Ca² ⁺ responses to CCL19 and CCL21 in cells expressing each CCR7 variant under UbiC control. Data represent the mean ± SEM of three independent experiments. *p < 0.05, **p < 0.01 for CCL19 versus CCL21 (Student’s t-test or ANOVA with post hoc test).

Intracellular Ca² ⁺ mobilization was monitored in Gqi-expressing HEK293 cells using NanoBiT-based Ca² ⁺ probes. Under CMV control, both CCR7A and CCR7C mediated strong Ca² ⁺ responses to 100 ng/ml CCL19, with somewhat weaker but still robust responses to CCL21 (Fig 3A). Under UbiC-driven expression, CCR7A still produced strong Ca² ⁺ mobilization in response to both CCL19 and CCL21, comparable in amplitude to CMV-driven expression. In contrast, UbiC-driven CCR7C produced only modest Ca² ⁺ responses to CCL19 and virtually no response to CCL21, despite detectable surface expression (Fig 3B). CCR7B failed to induce Ca² ⁺ increases under either promoter (Fig 3A, B).

Dose–response curves for UbiC-driven CCR7A yielded EC_50_ values of ~4.5 × 10^-9^ M for CCL19 and ~1.7 × 10^-8^ M for CCL21, confirming that CCL19 shows higher potency and efficacy (Fig 3C, left graph). For UbiC-driven CCR7C, an EC_50_ of ~5.7 × 10^-9^ M could be determined for CCL19, but reliable EC_50_ estimation for CCL21 was not possible due to weak responses (Fig 3C, right graph). With regard to maximal Ca² ⁺ responses, CCL19 elicits higher responses in both isoforms (Fig 3D). These results indicate that CCR7A is the major functional receptor isoform under controlled expression conditions possibly similar to endogenous expression, whereas CCR7C is a weaker, CCL19-biased receptor.

### β-arrestin/GRK signaling and dimerization of CCR7 isoforms

**Fig 4:**
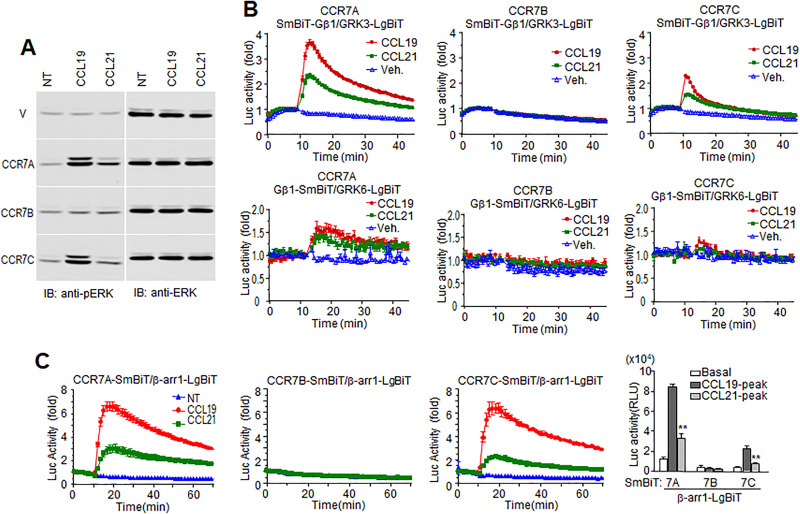
Chemokine-stimulated ERK phosphorylation and GRK/β-arrestin recruitment to CCR7 variants. (A) HEK293 cells expressing CCR7A, CCR7B, or CCR7C were stimulated with 100 ng/ml CCL19 or CCL21 for 5 min. Cell lysates were analyzed by Western blotting with anti-phospho-ERK (pERK) antibodies. The protein amount loaded into each well was normalized by probing with total ERK antibodies. (B) Chemokine-induced interactions between Gβ1 and GRKs. Upper panels: cells co-expressing SmBiT-Gβ1, GRK3-LgBiT, and each CCR7 variant were stimulated with CCL19 or CCL21, and luminescence was recorded over time. Lower panels: cells co-expressing Gβ1-SmBiT, GRK6-LgBiT, and each CCR7 variant were analyzed similarly. (C) β-arrestin1 recruitment to CCR7 variants measured by NanoBiT. HEK293 cells co-expressing β-arrestin1-LgBiT and C-terminal SmBiT-tagged CCR7A, CCR7B, or CCR7C were stimulated with CCL19 or CCL21, and luminescence was monitored over time. Line graphs show time-courses of luminescence; bar graphs depict peak luminescence values for each condition. **p < 0.01 for CCL19 versus CCL21.

We next examined ERK phosphorylation, GRK recruitment, and β-arrestin1 binding. In HEK293 cells expressing CCR7A, CCL19 induced robust ERK1/2 phosphorylation, whereas CCL21 caused a weaker increase. CCR7C-expressing cells showed a detectable pERK response to CCL19 but little or no response to CCL21, and no ligand-induced ERK phosphorylation was observed in CCR7B-expressing or control cells (Fig 4A). Gβ1–GRK interactions were monitored using NanoBiT assays with SmBiT-tagged Gβ1 and LgBiT-tagged GRK3 or GRK6. In CCR7A-expressing cells, both CCL19 and CCL21 significantly increased Gβ1–GRK3 interaction, with CCL19 producing slightly higher signals (Fig 4B, upper graphs). Ligand-induced Gβ1–GRK6 interactions were weaker, suggesting that GRK3 may be more prominently engaged by CCR7 under our assay conditions (Fig 4B, lower graphs). However, it cannot be concluded that CCR7 preferentially interacts with the Gβ1–GRK3, because the Gβ1 interaction configurations used for GRK3 and GRK6 in the NanoBiT assay were different (SmBiT-Gβ1 for GRK3-LgBiT, Gβ1-SmBiT for GRK6-LgBiT), potentially leading to distinct luminescence signals independent of the actual interaction strength. CCR7C showed a similar pattern but with reduced signal amplitude, whereas CCR7B did not support ligand-dependent Gβ1–GRK interactions.

β-arrestin1 recruitment was examined by co-expressing β-arrestin1-LgBiT with SmBiT-tagged CCR7 isoforms. CCR7A displayed strong, time-dependent increases in luminescence in response to CCL19 and, to a lesser extent, CCL21. CCR7C also recruited β-arrestin1 in response to CCL19, but the maximal increases and absolute luminescence values were lower than those observed for CCR7A, and CCL21-induced responses were minimal. CCR7B did not exhibit detectable ligand-dependent β-arrestin1 recruitment. Together with the Ca²⁺ and mini-G data, these results further support CCR7A as the dominant functional isoform and CCR7C as a weaker, CCL19-biased receptor (Fig 4C).

**Fig 5:**
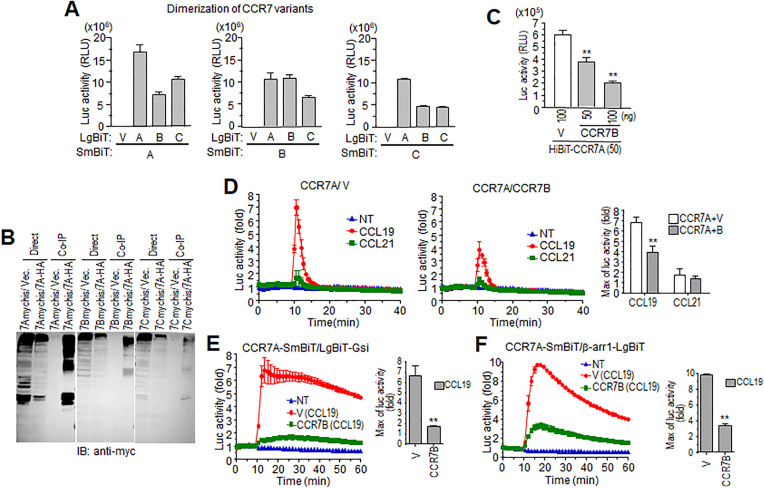
Dimerization of CCR7 variants and inhibitory effects of CCR7B on CCR7A expression and signaling. (A) NanoBiT-based analysis of homo- and heterodimerization among CCR7 isoforms. HEK293 cells were co-transfected with each combination of C-terminal LgBiT-tagged and C-terminal SmBiT-tagged CCR7A, CCR7B, or CCR7C. The luminescence in normal culture condition was measured to determine receptor-receptor association. Data represent the mean ± SEM of three independent experiments. (B) Co-immunoprecipitation of CCR7 isoforms. HEK293 cells co-expressing C-terminal HA-tagged CCR7A and each C-terminal myc-his–tagged CCR7 isoform were subjected to immunoprecipitation with anti-HA agarose. Precipitates were analyzed by Western blotting with anti-myc antibody to detect associated CCR7 variants. (C) Effect of CCR7B on cell surface expression of CCR7A. HEK293 cells were co-transfected with HiBiT-CCR7A and increasing amounts of CCR7B plasmid. Extracellular HiBiT activity was quantified 24 h after transfection. Data represent the mean ± SEM; **p < 0.01 versus vector control. (D) Effect of CCR7B on chemokine-induced Ca² ⁺ responses. Gqi-expressing HEK293 cells were transfected with CCR7A alone or together with CCR7B and stimulated with CCL19 or CCL21; Ca² ⁺ -dependent luminescence was recorded over time. Data represent mean ± SEM from three independent experiments. Bar graph shows maximum luciferase activities induced by the chemokines. **p < 0.01 for CCR7A/B versus CCR7A alone. NT, no treatment. (E) Effect of CCR7B on CCL19-induced interaction between CCR7A and mini-Gi. Cells co-expressing CCR7A-SmBiT, LgBiT-mini-Gsi, and either vector or CCR7B were stimulated with CCL19, and luminescence was measured. Bar graph shows maximum activities by CCL19. **p < 0.01 for CCR7B versus vector. (F) Effect of CCR7B on CCL19-induced β-arrestin1 recruitment to CCR7A. Cells co-expressing CCR7A-SmBiT, β-arrestin1-LgBiT, and either vector or CCR7B were stimulated with CCL19, and NanoBiT luminescence was recorded. Bar graph shows maximum activities by CCL19. **p < 0.01 for CCR7B versus vector.

To assess isoform–isoform interactions, we performed NanoBiT assays with all pairwise combinations of C-terminal SmBiT- and LgBiT-tagged CCR7A, CCR7B, and CCR7C. All combinations yielded luminescence signals above vector controls, indicating that each isoform can form homodimers and heterodimers (Fig 5A). Combinations involving CCR7A generally produced higher signals, likely reflecting better expression and membrane localization. Co-immunoprecipitation using HA-tagged CCR7A and myc-tagged CCR7A/B/C confirmed that CCR7A can interact with all three isoforms biochemically (Fig 5B).

### CCR7B acts as a dominant-negative regulator and limits chemokine-induced migration

Given that CCR7B was non-signaling but capable of forming heterodimers with CCR7A, we hypothesized that CCR7B might modify CCR7A function. Co-expression of CCR7B with HiBiT-CCR7A led to a dose-dependent reduction in cell surface HiBiT signal, indicating reduced CCR7A surface expression (Fig 5C). Functionally, CCR7B co-expression significantly diminished CCR7A-mediated Ca² ⁺ responses to both CCL19 and CCL21 (Fig 5D). The CCL19-induced interactions between CCR7A-SmBiT and mini-Gsi-LgBiT, and between CCR7A-SmBiT and β-arrestin1-LgBiT, were likewise attenuated by CCR7B, demonstrating a dominant-negative effect on both G protein- and β-arrestin-dependent pathways (Fig 5E, F).

**Fig 6:**
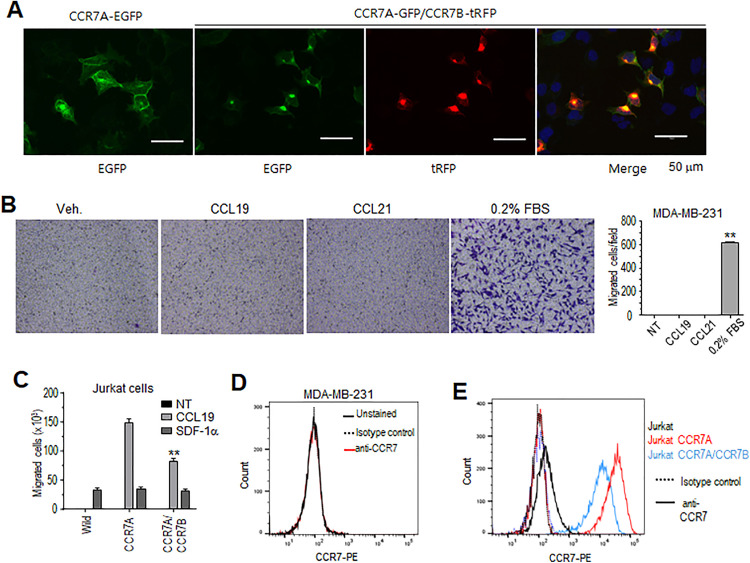
CCR7B inhibits membrane localization of CCR7A and chemokine-induced migration. (A) HEK293 cells were co-transfected with CCR7A-EGFP and CCR7B-tRFP. Fluorescence signals were observed by confocal microscopy. Note the reduction of CCR7A-EGFP at the plasma membrane and its redistribution to intracellular compartments where CCR7B-tRFP is enriched. Scale bar, 50 μm. (B) Chemotactic migration of MDA-MB-231 cells in response to CCL19, CCL21, or 0.2% FBS. Cells were subjected to transwell migration assays, and cells that migrated to the lower surface of the membrane were fixed, stained, and counted under a light microscope. Bar graph shows the number of migrated cells per field (mean ± SEM); **p < 0.01 versus No treatment (NT). (C) Jurkat cells expressing exogenous CCR7A or CCR7A/B were subjected to transwell migration assay with CCL19. After 6 h incubation, the cells that migrated to the lower chamber were collected and counted. Data represent the mean ± SEM; **p < 0.01 versus CCR7A alone. (D) Cell-surface CCR7 expression in MDA-MB-231 cells. Trypsinized MDA-MB-231 cells were stained with PE-conjugated anti-human CCR7 antibody or PE-conjugated isotype control and analyzed by flow cytometry. (E) Flow cytometric analysis of cell-surface CCR7 in Jurkat cells. Parental Jurkat cells, Jurkat cells transduced with CCR7A, and Jurkat cells co-transduced with CCR7A and CCR7B were stained with PE-conjugated anti-human CCR7 antibody or isotype control and analyzed by flow cytometry to assess surface CCR7 expression.

Confocal imaging of cells co-expressing CCR7A-EGFP and CCR7B-tRFP showed that CCR7A-EGFP, which localized mainly at the plasma membrane when expressed alone, redistributed to intracellular compartments in the presence of CCR7B, where it strongly co-localized with CCR7B-tRFP. These observations suggest that CCR7B retains CCR7A in intracellular compartments and prevents efficient delivery to, or stability at, the plasma membrane (Fig 6A).

To explore the potential relevance of this mechanism in cancer cells, we examined chemokine-induced migration of MDA-MB-231 breast cancer cells, which express CCR7A and CCR7B transcripts. In transwell assays, neither CCL19 nor CCL21 induced significant migration relative to vehicle. In contrast, 0.2% FBS induced robust migration, demonstrating that these cells are intrinsically motile and capable of responding to chemotactic stimuli. The absence of chemokine-induced migration despite CCR7A/B expression suggests that CCR7 signaling is functionally suppressed in these cells, potentially due to the dominant-negative effect of CCR7B on CCR7A (Fig 6B).

Jurkat cells did not migrate toward CCL19 in the Transwell assay, even though they are T-lymphocyte–derived and expressed CCR7 splice variant mRNAs by RT-PCR. To assess whether CCR7A is sufficient to confer CCL19 responsiveness, we reconstituted Jurkat cells with exogenous CCR7A or with both CCR7A and CCR7B using lentiviral transduction and subjected them to Transwell migration assays. Jurkat cells expressing CCR7A alone showed robust migration toward CCL19, whereas CCL19-induced migration was significantly reduced when CCR7A and CCR7B were co-expressed (Fig 6C). Consistent with these findings, flow cytometric staining with a PE-conjugated anti-CCR7 antibody showed minimal endogenous CCR7 at the surface of Jurkat and MDA-MB-231 cells, whereas lentiviral expression of CCR7A in Jurkat cells produced a robust CCR7 signal. Co-expression of CCR7B with CCR7A reduced the CCR7-positive population and mean fluorescence intensity compared with CCR7A alone, supporting the notion that CCR7B diminishes functional CCR7A at the plasma membrane in T cells as well as in heterologous HEK293 systems (Fig 6D and E).

## Discussion

In this study, we performed an integrated characterization of three human CCR7 variants generated by alternative splicing and showed that they differ markedly in trafficking, signaling capacity, and mutual interactions. The data support a model in which CCR7A serves as the principal functional receptor for CCL19 and CCL21, which is consistent with previous reports [28,29]. CCR7C functions as a weak and predominantly CCL19-responsive isoform with poor membrane targeting, and CCR7B acts as a non-signaling dominant-negative isoform that suppresses CCR7A-mediated signaling.

A central feature of our work is the combined use of strong (CMV) and weaker (UbiC) promoters to control receptor expression. CMV-driven gene expression has been extensively used in various biological assays using exogenous protein expression. While high-level expression is convenient and can reveal maximal signaling potential, it often exaggerates receptor function and masks subtle but biologically relevant differences between isoforms [24]. By complementing CMV-driven assays with UbiC-driven expression, we were able to demonstrate that CCR7A retains strong signaling under controlled expression levels, whereas CCR7C and CCR7B remain weak or non-functional. Under UbiC control, CCR7A mediated robust Gi/o activation, Ca² ⁺ mobilization, ERK phosphorylation, GRK recruitment through Gβ, and β-arrestin1 binding with nanomolar sensitivity to CCL19 and CCL21, whereas CCR7C showed only modest responses to CCL19 and only a very weak response to CCL21, and CCR7B did not respond to either ligand.

Our findings highlight the importance of the N-terminal region and signal peptide in CCR7 biology. As a class A type GPCR, most chemokine receptors do not carry a signal peptide in the N-terminal extracellular region, because part of the N-terminus together with the first transmembrane domain function as an uncleavable signal anchor to the ER membrane. The N-terminal region undergoes post-translational modification and is pivotal for ligand binding [30,31]. Interestingly, both CCR7A and CCR7C possess signal peptides with different numbers of amino acids [23]. CCR7A’s longer signal sequence and favorable Kozak context correlate with efficient plasma-membrane localization and strong signaling. CCR7C, which has a shorter signal peptide and a suboptimal start site, accumulates in perinuclear compartments and has reduced surface expression and signaling, particularly in response to CCL21. Importantly, because the signal peptide is cleaved co-translationally, the mature CCR7C protein is identical in sequence to mature CCR7A. CCR7C is therefore not expected to act as a dominant-negative regulator; rather, any CCR7C that successfully reaches the plasma membrane would likely function equivalently to CCR7A. The quantitative difference in surface expression and signaling between CCR7A and CCR7C thus reflects differences in biosynthetic efficiency rather than in the intrinsic signaling properties of the mature receptor. CCR7B lacks a canonical N-terminal extracellular domain and signal peptide, is largely retained intracellularly, and does not couple to G proteins or recruit β-arrestin1. These observations are consistent with studies of other chemokine receptors where 5′ alternative splicing alters N-terminal structure and modulates membrane expression and ligand responsiveness [24,32].

The most distinctive property of CCR7B is its ability to modulate CCR7A function despite lacking intrinsic signaling capacity. Our NanoBiT and co-immunoprecipitation data show that CCR7B forms heterodimers with CCR7A, and functional assays demonstrate that CCR7B reduces CCR7A surface expression and attenuates CCR7A-mediated Ca² ⁺ responses, mini-Gi interaction, and β-arrestin1 recruitment. Imaging further reveals that CCR7B redistributes CCR7A from the plasma membrane to intracellular compartments. Together, these findings support a dominant-negative role for CCR7B, likely through intracellular retention or mislocalization of CCR7A. Similar dominant-negative effects have been reported for splice variants of other GPCRs, suggesting that non-signaling isoforms may function as endogenous brakes on receptor activity [33,34]. Although CCR7B-mediated suppression of CCR7C was not directly examined, the near-identical transmembrane and intracellular sequences shared between CCR7A and CCR7C suggest that CCR7B would similarly reduce CCR7C surface expression and signaling; however, given CCR7C’s already limited membrane targeting and minor expression level, this effect is expected to be of lesser physiological consequence.

Our results in MDA-MB-231 breast cancer cells suggest that the balance of CCR7 splice variants, rather than total CCR7 expression, may determine chemokine responsiveness in tumors. There are reports demonstrating functional roles of CCR7 in epithelial-mesenchymal transition, migration, and invasion of breast cancer cells, including MDA-MB-231 [35–37]. These cells express both CCR7A and CCR7B transcripts as determined by RT-PCR; however, under our experimental conditions they did not migrate toward CCL19 or CCL21, while they readily migrated toward serum-containing medium. One possible explanation is that high CCR7B expression restricts functional CCR7A at the cell surface, thereby blunting in vitro chemokine-driven migration. This may help explain why CCR7 mRNA or protein expression in tumors does not always correlate with measurable chemokine-induced migration. Endogenous CCR7 protein is often barely detectable with some commercial antibodies, either because its abundance in the plasma membrane is low or because of suboptimal antibody performance. In line with these limitations, we focused on flow cytometry as a more sensitive and direct approach to quantify cell-surface CCR7 in Jurkat and MDA-MB-231 cells, rather than relying on conventional Western blotting of endogenous GPCRs. Nonetheless, future studies that quantify each isoform at the protein level in patient samples and correlate isoform ratios with lymph node metastasis and clinical outcomes will be important to test this hypothesis. The dominant-negative effect of CCR7B was further confirmed by its inhibition of CCR7A-mediated migration toward CCL19 in Jurkat T cells exogenously expressing CCR7 isoforms. Consistent with this model, flow cytometric analysis using anti-CCR7 antibodies showed that CCR7 is barely detectable at the plasma membrane of both MDA-MB-231 and parental Jurkat cells, whereas lentiviral CCR7A expression increases surface CCR7 in Jurkat cells and CCR7B co-expression reduces this increase. These data support the view that CCR7B primarily limits the surface-accessible pool of CCR7A and help explain why CCR7 transcript or total protein levels do not necessarily predict chemokine-induced migration.

As a limitation of this study, most experiments used heterologous HEK293-based systems and engineered Gqi-expressing cells rather than primary immune cells or in vivo models, so the physiological contributions of each isoform remain to be established. Isoform-level quantification of CCR7 transcripts in primary naive T and B cells using long-read sequencing approaches will be important to determine whether the CCR7A/CCR7B-predominant pattern observed in transformed Jurkat and Raji cells faithfully reflects the physiological splice variant balance in primary lymphocytes. We did not directly dissect the intracellular compartments or pathways by which CCR7B retains or redirects CCR7A, nor did we analyze other long-term functional consequences of isoform expression such as gene expression changes or cell differentiation. Additionally, we did not examine how inflammatory cues or tumor microenvironmental factors might alter the expression balance among CCR7 isoforms. Despite these limitations, our work provides a framework for understanding how CCR7 splice variants collectively shape chemokine responses. It suggests that tuning the relative expression of CCR7A, CCR7B, and CCR7C may offer a more efficient strategy to modulate CCR7-dependent processes than global receptor blockade. For example, in conditions where excessive CCR7 signaling contributes to pathology, enhancing CCR7B expression or mimicking its dominant-negative effect might attenuate CCR7A activity without completely abolishing immune surveillance. Conversely, in tumors where CCR7A-driven lymph node metastasis is supported by low CCR7B expression, selective targeting of CCR7A or restoration of CCR7B function could help limit metastatic spread.

In conclusion, human CCR7 alternative splicing yields a functional receptor (CCR7A), a weak CCL19-biased isoform (CCR7C), and a non-signaling dominant-negative isoform (CCR7B) that together fine-tune chemokine responsiveness. By analyzing these isoforms under controlled expression conditions and employing highly sensitive assays to probe receptor–ligand interactions and downstream signaling, we delineate an additional layer of CCR7 regulation with potential significance for immune homeostasis and cancer biology.

## Supporting information

S1 File Raw images. (PDF)

S2 File Raw data. (ZIP)

## Abbreviations

CCR7: C-C chemokine receptor type 7
CCL19/21: C-C motif chemokine ligand 19/21
CMV: cytomegalovirus
UbiC: ubiquitin C
CXCR: C-X-C chemokine receptor
EGFP: enhanced green fluorescent protein
tRFP: turbo red fluorescent protein
ERK: extracellular signal-regulated kinase
GPCR: G protein-coupled receptor
GRK: G protein-coupled receptor kinase
HA: hemagglutinin epitope tag
HRP: horseradish peroxidase
