# Supplementary material for: Functional CCR7A-mediated cellular responses are negatively modulated by the splice variant CCR7B
Source: PLoS One. 2026 Aug 4;21(8):e0354819. doi: 10.1371/journal.pone.0354819 (PMC13436775; doi:10.1371/journal.pone.0354819)
Supplement: Supplementary file 1 [file pone.0354819.s001.pdf]

Fig 1C

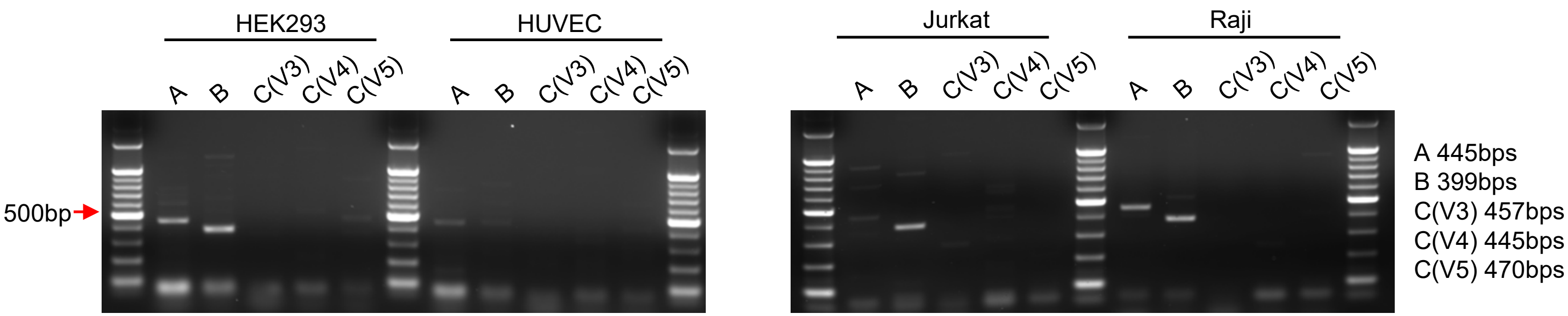


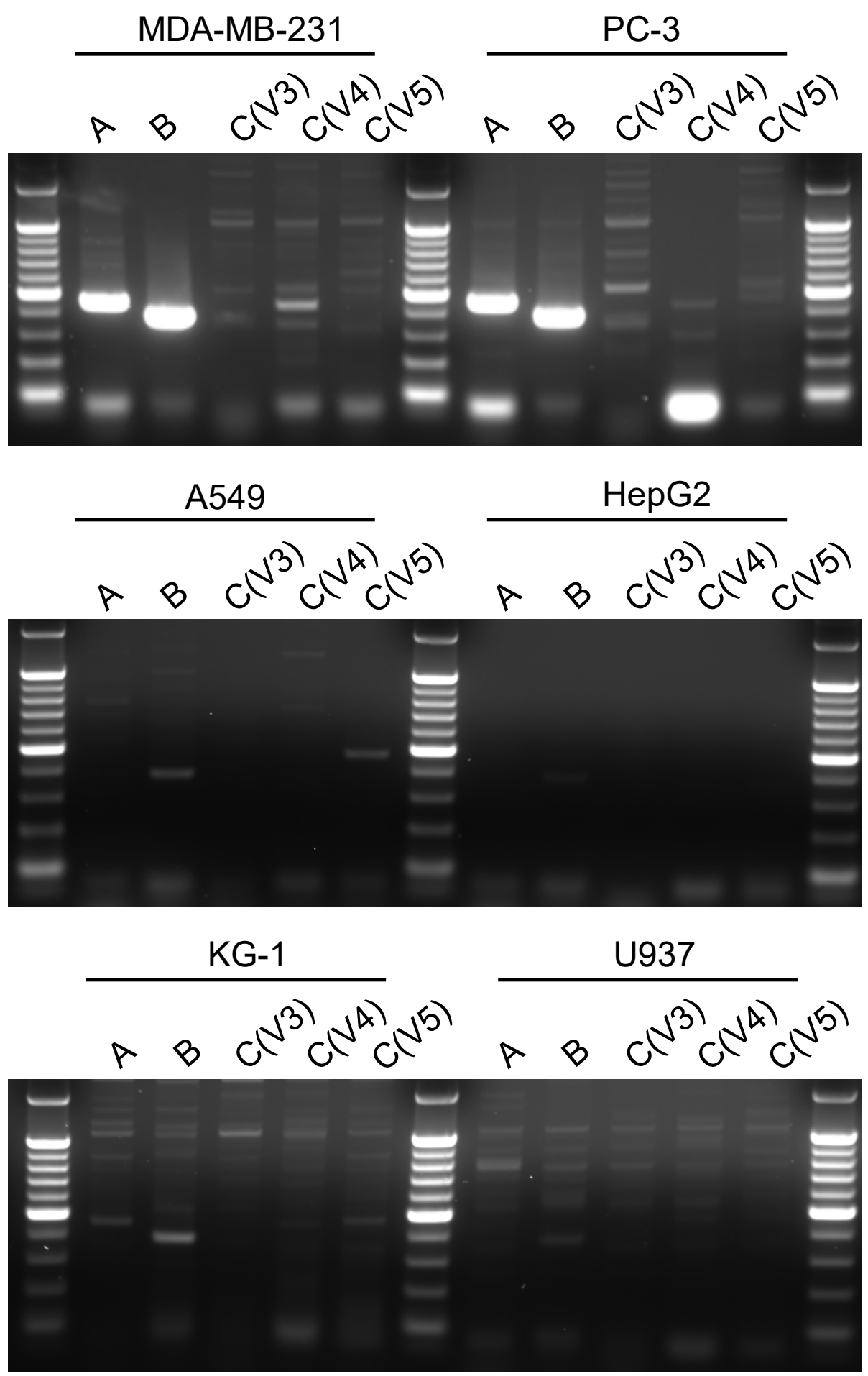


Fig 1E

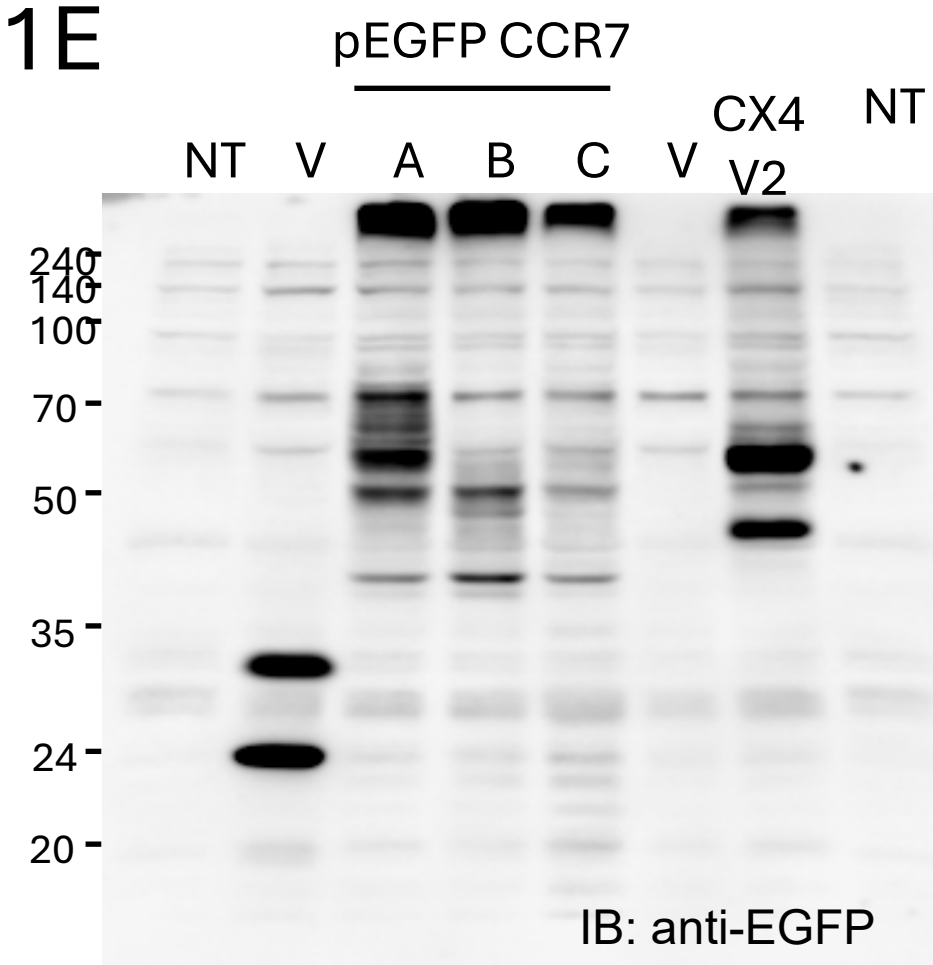


Fig 1F

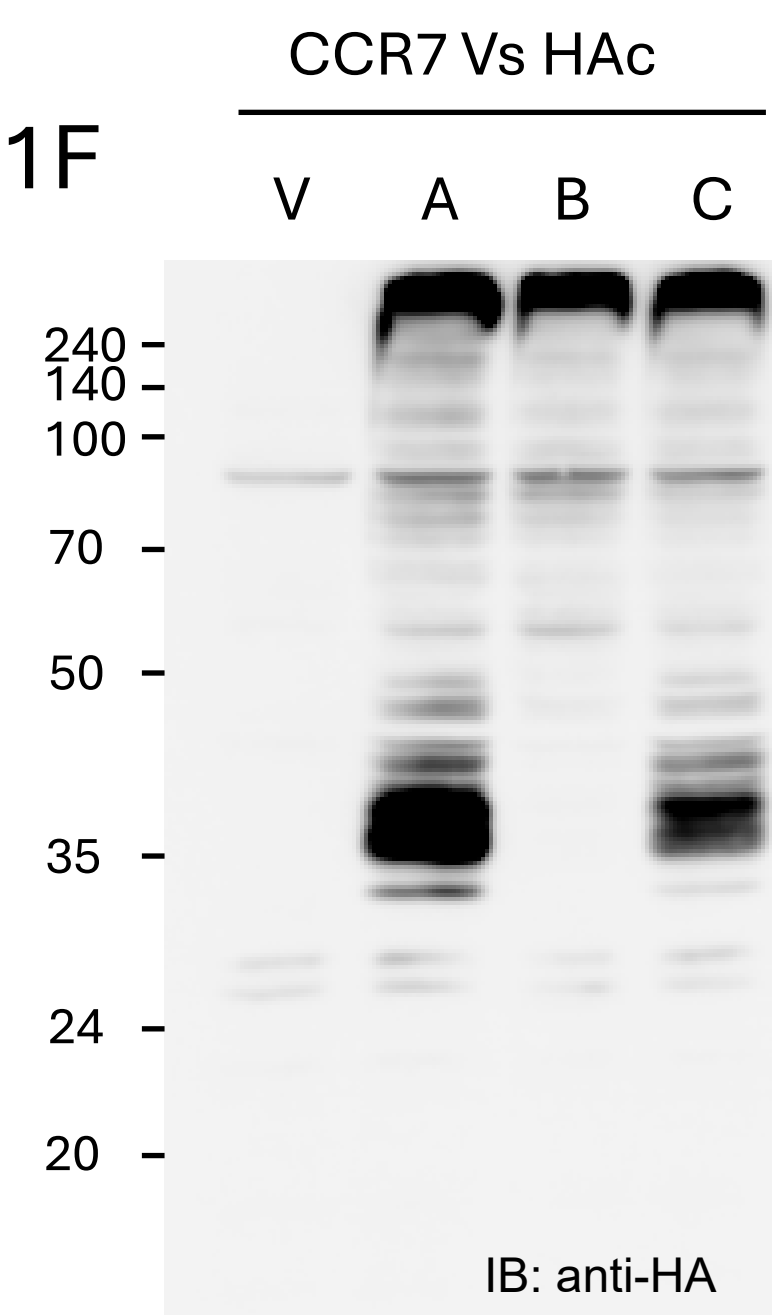

Fig 4A

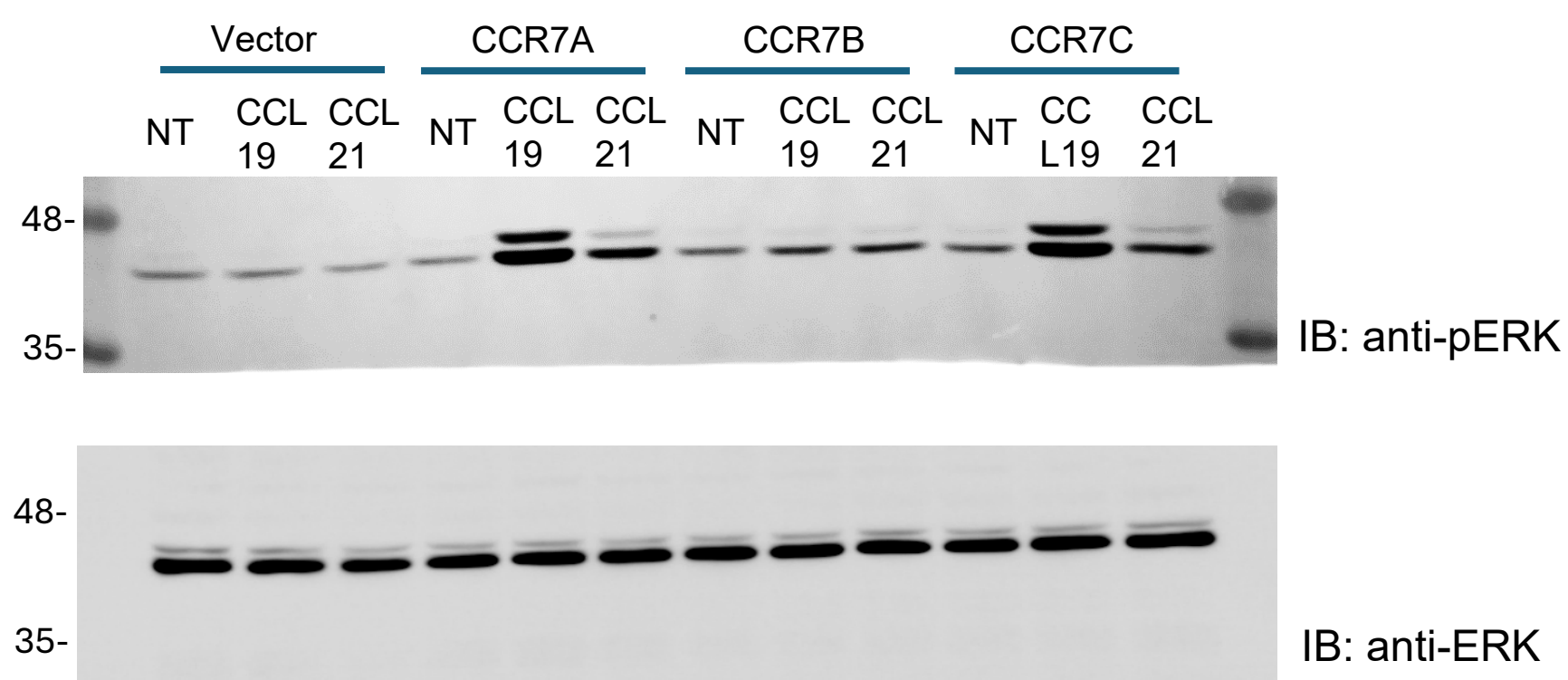

Vector
CCR7A
CCR7B
CCR7C
NT
CCL 19
CCL 21
NT
CCL 19
CCL 21
NT
CCL 19
CCL 21
NT
CC L19
CCL 21
48-
35-
IB: anti-pERK
48-
35-
IB: anti-ERK


Fig 5A

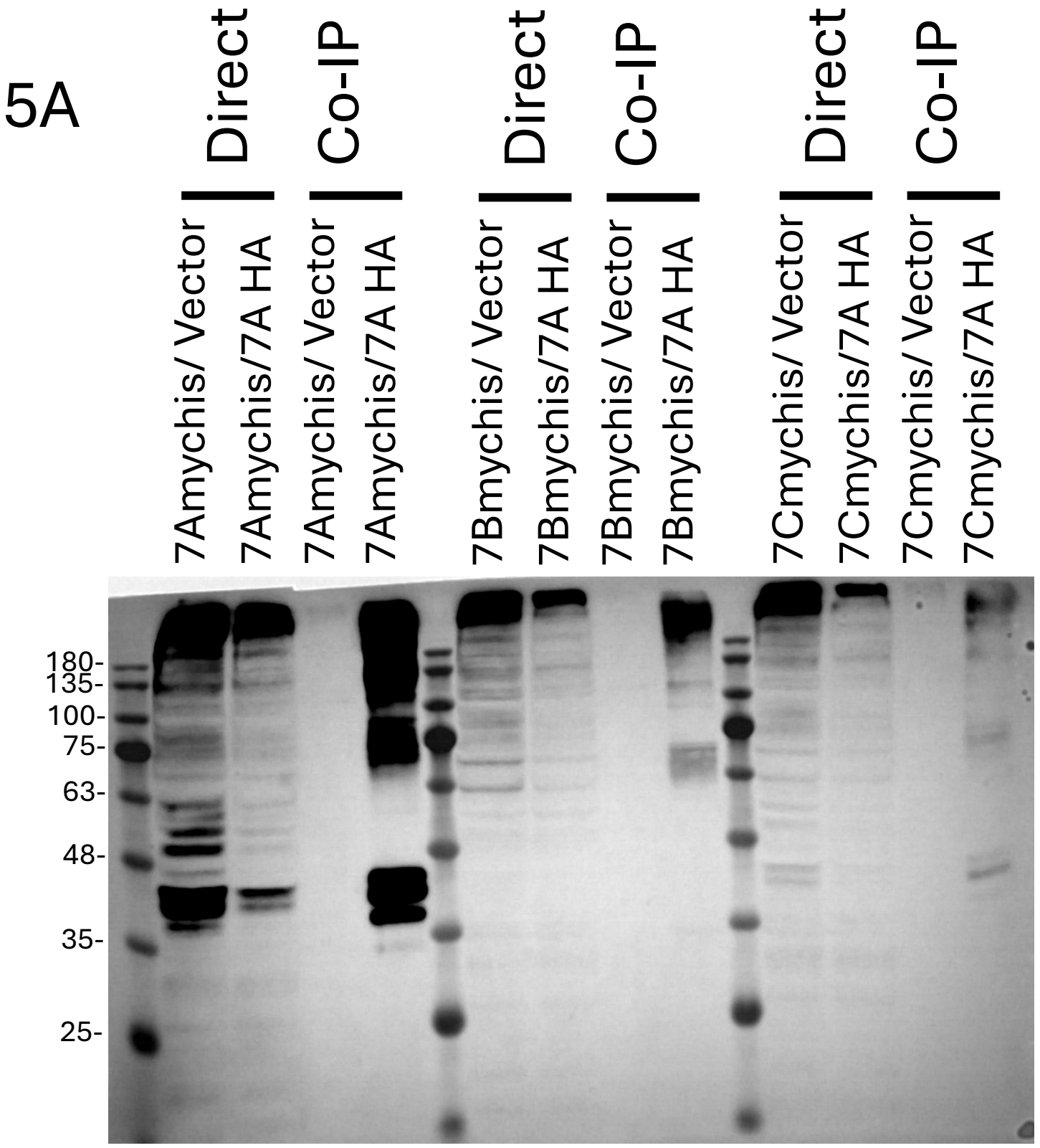

Direct
Co-IP
Direct
Co-IP
Direct
Co-IP
7Amychis/ Vector
7Amychis/7A HA
7Amychis/ Vector
7Amychis/7A HA
7Bmychis/ Vector
7Bmychis/7A HA
7Bmychis/ Vector
7Bmychis/7A HA
7Cmychis/ Vector
7Cmychis/7A HA
7Cmychis/ Vector
7Cmychis/7A HA
180-
135-
100-
75-
63-
48-
35-
25-
